# SNIT: SNP identification for strain typing

**DOI:** 10.1186/1751-0473-6-14

**Published:** 2011-09-08

**Authors:** Ravi Vijaya Satya, Nela Zavaljevski, Jaques Reifman

**Affiliations:** 1 Biotechnology HPC Software Applications Institute, Telemedicine and Advanced Technology Research Center, U.S. Army Medical Research and Materiel Command, Fort Detrick, MD 21702, USA

## Abstract

With ever-increasing numbers of microbial genomes being sequenced, efficient tools are needed to perform strain-level identification of any newly sequenced genome. Here, we present the SNP identification for strain typing (SNIT) pipeline, a fast and accurate software system that compares a newly sequenced bacterial genome with other genomes of the same species to identify single nucleotide polymorphisms (SNPs) and small insertions/deletions (indels). Based on this information, the pipeline analyzes the polymorphic loci present in all input genomes to identify the genome that has the fewest differences with the newly sequenced genome. Similarly, for each of the other genomes, SNIT identifies the input genome with the fewest differences. Results from five bacterial species show that the SNIT pipeline identifies the correct closest neighbor with 75% to 100% accuracy. The SNIT pipeline is available for download at http://www.bhsai.org/snit.html

## Background

Rapid and accurate identification of an infectious agent is of the utmost importance for the surveillance and treatment of infectious diseases. Traditionally, strain typing has been performed using assays that probe a few previously known polymorphic loci [1]. However, due to the inherent limitations of using only a few loci, these methods offer low specificity.

Because of the rapid decrease in costs of genome sequencing, strain typing can now be performed *in silico *by first sequencing the sample, and then comparing the genome sequence with other available genomes of the same species to identify the closest strain. This approach has the potential to offer a much higher specificity because it uses the entire genome rather than a few pre-selected loci. Moreover, a comprehensive listing of all polymorphisms in a newly sequenced genome might also be useful in predicting the virulence or pathogenicity of the new strain.

Single nucleotide polymorphisms (SNPs) are the most abundant form of genetic variation. Many previous methods have used "in-house" pipelines to identify and catalog the SNPs between pathogens of the same species [2,3]. However, with the exception of SNPsFinder [4] and inGAP [5], these pipelines are seldom publicly available. The SNPsFinder pipeline is a Web-based application that requires users to upload the genome sequences that need to be compared, which might be time consuming when a large number of genomes are involved. In addition, the use of a public server is not desirable if confidentiality of the data is a concern. The inGAP pipeline provides many useful functionalities for the analysis of next-generation sequencing data, however, the SNP identification routines do not scale well with the number of genomes because of their reliance on multiple sequence alignments. In our comparative investigation, inGAP successfully produced SNPs for four *Shigella flexneri *genomes, but repeatedly crashed when run for seven *Burkholderia mallei *genomes (the Results Section contains details of the configuration of the systems on which these comparisons were performed).

Here, we present the SNP Identification for Strain Typing (SNIT) pipeline, a computationally efficient, light-weight application that analyzes multiple genomes and identifies SNPs and small indels. The pipeline has many advantages: *1*) it is a stand-alone application with a graphical user interface (GUI) that runs on the user's workstation, thus eliminating issues of data confidentiality; *2*) it is accurate, fast, and highly scalable, owing to the use of pairwise alignments to achieve the basic functionality of SNP finding; and *3*) it automatically identifies the closest neighbor for each genome without the need for manual processing of the SNP data.

## Implementation

**Figure 1:**
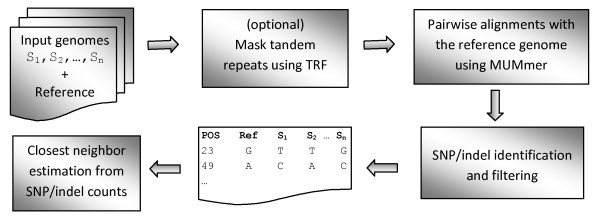
**Outline of the SNP identification pipeline**. The tandem repeat regions in the input genomes can be masked using the Tandem Repeat Finder (TRF) program. Each input genome is aligned against a user-specified reference genome. The lists of differentiating SNPs and indels between each pair of input genomes are constructed from these pairwise alignments.

The input to the pipeline can be any combination of complete genomes or draft assemblies. Optionally, the user can also provide quality scores for draft assemblies to enable masking low-quality bases and ignoring the SNPs reported at these positions. In the first step, the tandem repeat regions in the input genomes are masked to avoid reporting ambiguous variations from these regions. We used the program Tandem Repeat Finder (TRF) [6] to mask these tandem repeat regions. In the second step, SNIT performs pairwise alignments between each input genome and a user-selected reference genome (from the list of input genomes) using the *nucmer *program of the MUMmer software [7]. SNIT uses the *delta-filter *utility of MUMmer to filter these alignments and obtain a one-to-one mapping between the query and the reference. The pipeline then processes these filtered alignments to tabulate a list of SNPs and small indels. Figure 1 shows a high-level outline of the pipeline.

The polymorphisms from the individual pairwise alignments are then combined into a single table that contains the position of each polymorphism in the reference genome and the individual variants in each of the input genomes. In compiling these tables, any position in the query genome that is not part of a filtered alignment with the reference is considered as missing (i.e., part of a large insertion or deletion) in the query genome. Various filters can be applied in building this table, including requirements on the length of conserved sequence on either side of a polymorphism and the selection of only those polymorphisms that are present in all input genomes. The numbers of differentiating SNP/indel loci between each pair of input genomes are computed by comparing the corresponding columns in this table. For each input genome, the pipeline analyzes the polymorphic loci present in all input genomes and reports the genome with the fewest differences as the closest neighbor.

## Results

### Accuracy and efficiency with draft and complete genomes

We tested the accuracy and efficiency of the SNIT pipeline for five different bacterial species. For each species, we ran SNIT using all publicly available strains that were included in published phylogenies [3,8-11], including strains for which only draft genomes were available. These phylogenies were used to estimate the accuracy of the SNIT pipeline. Table 1 lists the input parameters used in these comparisons.

**Table 1:** Input parameters used for testing SNIT

Parameter	Value
Minimum MUMmer cluster length	100
Minimum MUMmer exact match	50
Maximum MUMmer gap	49
Minimum large indel size	50
Minimum conserved flank length	50

Table 2 summarizes the results for the five bacterial species. The SNIT pipeline took < 2 min to compare four *S. flexneri *genomes. In contrast, the SNPsFinder pipeline took 20 min, and the multiple genome comparison module of inGAP took 31 min. The SNIT pipeline was able to efficiently process a large number of input genomes, taking only 45 min to compare 20 large *Burkholderia pseudomallei *genomes, while the SNPsFinder and inGAP pipelines repeatedly failed to produce any results for this test case. Overall, the SNP pipeline scales linearly with the number of input genomes, as each genome is only compared against the selected reference genome.

**Table 2:** Summary of the results for five different bacterial species

Species	No. of Genomes	Combined Size (Mbp)	Time (min)	Accuracy (%)
*Bacillus anthracis*	7	36	4	100
*Francisella tularensis*	11	22	3	100
*Shigella flexneri*	4	18	2	100
*Burkholderia mallei*	10	59	19	100
*Burkholderia pseudomallei*	20	144	45	75

Accuracy is defined as the percentage of genomes for which the correct closest neighbors were identified based on published phylogenies for the species. The runs were performed using a single processor on a 3.6 GHz dual processor system with 4 GB RAM.

The SNIT pipeline correctly identified the closest neighbors for 100% of the genomes in four out of the five test cases, including clonal species, such as *Bacillus anthracis *and *Francisella tularensis*. For the fifth test case, *B. pseudomallei*, the accuracy was 75% (15 out of 20). The lower accuracy for *B. pseudomallei *is not surprising, because the strains of this species are highly divergent, with horizontal transfer playing a significant role in their evolutionary history. A more sophisticated approach than simple SNP and indel counts would be necessary for accurate typing of such highly divergent species as *B. pseudomallei*. The details of the genomes and phylogenies used in these comparisons are provided in Additional file 1.

**Figure 2:**
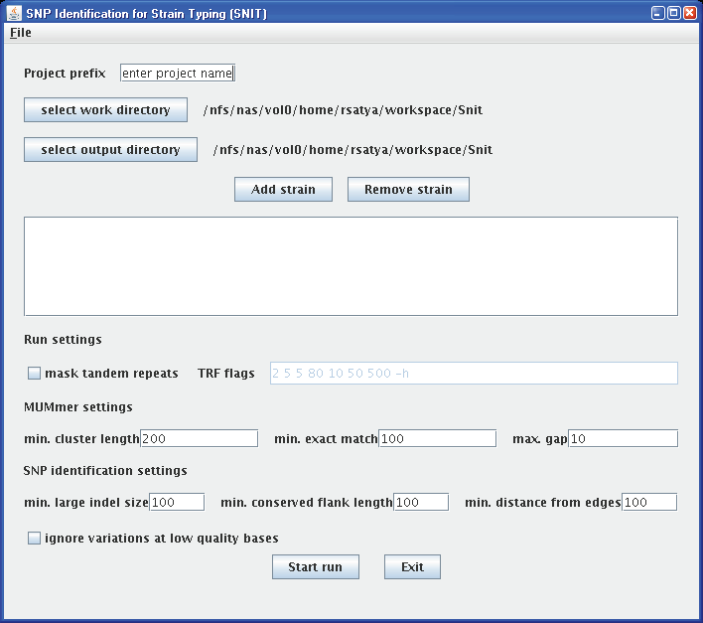
**Graphical user interface of the SNIT pipeline**. The screenshot of the main interface, showing the default values of the various input parameters.

The SNIT pipeline provides a GUI that allows users to select the input genomes, settings, and run the SNP identification pipeline. Figure 2 provides a screenshot of the GUI.

### Accuracy with next-generation sequencing data

To test the applicability of SNIT to assemblies generated from next-generation sequencing (NGS) data, we selected the recently published *Yersinia pestis *KIM D27 genome [12]. The *Y. pestis *D27 strain is a derivative of *Y. pestis *KIM 10 strain (accession no. NC_004088). The *Y. pestis *KIM D27 draft genome (accession no. ADDC00000000) was generated from a hybrid assembly of reads generated from 454 XLR Titanium and Illumina Genome Analyzer IIx platforms. We configured a SNIT run with a total of 21 *Y. pestis *genomes, which included both draft and finished genomes. In the first run, we selected the *Y. pestis *KIM D27 draft genome as the reference. In this run, SNIT correctly identified the *Y. pestis *KIM 10 strain as the closest neighbor for *Y. pestis *KIM D27. Next, we repeated the run with *Y. pestis *CO92 selected as the reference genome. Again, SNIT correctly identified *Y. pestis *KIM 10 as the closest neighbor. These results suggest that the SNIT pipeline can be applied to assemblies generated from NGS data.

### Performance on larger data sets

To test the efficiency of the pipeline on even larger data sets, we ran SNIT with 50 arbitrarily selected *Escherichia coli *genomes downloaded from PATRIC [13]. For these 50 genomes, the pipeline completed the analysis (with the TRF option selected) in 145 min. However, nearly 110 of these 145 min were spent in running TRF on the input genomes. The pipeline completed the analysis in less than 32 min without the TRF option. These results indicate that the SNP pipeline can handle large data sets of 50 (or more) genomes.

## Discussion

In principle, the SNIT pipeline can be applied to contigs obtained from the sequencing of clinical samples, to perform strain-level identification of the pathogens present in the sample. The accuracy of such analysis will depend on the fraction of the target pathogen's genome covered by the contigs and the overall diversity among the different strains of the pathogen. However, the provided options to filter low-quality bases should reduce the effect of sequencing errors and, because SNIT's SNP identification is relative to the compared sequenced genomes, any remaining sequencing errors in the target sequence should not constitute a significant problem.

The efficiency of the SNIT pipeline stems from the use of pairwise alignments based on exact matches. However, this approach limits the application of the pipeline to bacterial and eukaryotic genomes. Due to the high variability in viral genomes, multiple genome alignments, possibly in the amino acid domain, will be necessary to identify discriminative polymorphisms for strain identification. Similar to other reference-based pairwise alignment approaches, such as SNPsFinder, the SNIT pipeline can only report SNP loci that can be mapped to the reference genome. While this capability is sufficient for strain typing, it should be noted that the pipeline is not intended to provide a comprehensive list of all SNPs among the input genomes. For instance, in the case of two genomes that share a large insertion compared with the reference genome, the variations within this large insertion would not be reported by SNIT unless one of them was selected as the reference. Hence, the SNIT pipeline is not ideal for use with strains with significant contributions from large insertions, deletions, or horizontal transfer events in their evolutionary history.

In general, we do not expect the performance of the pipeline to be drastically different on NGS data. While it is true that the error rate is higher for NGS data, sequencing errors should only have minimal, second-order effects on the overall results. This is because SNIT performs and reports the results of relative analysis, and it is highly unlikely that the same sequencing error would be repeated in two genomes, to make them appear closer than they should be. In addition, SNIT provides options to ignore variations at low-quality bases and at either end of contigs, which would help eliminate at least some of the sequencing errors from the analysis.

The results presented here indicate that the SNIT pipeline is highly accurate in identifying the closest neighbor even in cases of clonal species, such as *Bacillus anthracis*, *Francisella tularensis*, and *B. mallei*. Therefore, the pipeline can be useful as a rapid, automated tool for identifying the closest neighbor of a newly sequenced genome. The SNP identification modules from SNIT have been incorporated as part of the TOFI [14] and TOPSI [15] pipelines for designing pathogen diagnostic assays with strain-specific signatures.

## Availability and requirements

• **Project name: **SNIT

• **Project home page: **http://www.bhsai.org/snit.html

• **Operating systems: **Linux

• **Programming language: **Perl

• **Other requirements: **MUMmer 3.22 or greater, BioPerl, Tandem Repeat Finder (TRF) and Java Runtime Environment (JRE) 1.5 or greater

## Competing interests

The authors declare that they have no competing interests.

## Authors' contributions

RVS implemented the SNIT pipeline and the user interface. RVS and NZ analyzed the results from SNIT. JR provided overall project guidance. RVS and JR wrote the manuscript. All authors read and approved the final manuscript.

## Supplementary Material

Additional file 1 **Supplementary data**. Detailed results used to compute the accuracy for each of the five species detailed in Table 1 of the main text. Click here for file
