# Supplementary material for: SNIT: SNP identification for strain typing
Source: Source Code Biol Med. 2011 Sep 8;6:14. doi: 10.1186/1751-0473-6-14 (PMC3182885; doi:10.1186/1751-0473-6-14)
Supplement: Additional file 1 — Supplementary data. Detailed results used to compute the accuracy for each of the five species detailed in Table 1 of the main text. [file 1751-0473-6-14-S1.DOC]

Supplementary data for “SNIT: SNP identification for strain typing”

Ravi Vijaya Satya, Nela Zavaljevski, and Jaques Reifman

# Results

In the following, we present the detailed results used to compute the accuracy for each of the five species detailed in Table 1 of the main text. Although other sequenced strains were available for some of these species, we limited our analysis to the strains for which we could find published phylogenies.

For each species, we present the number of single nucleotide polymorphisms (SNP) or indel loci in which a pair of genomes have different alleles. This analysis is limited to the SNP/indel loci that are present in all the genomes of the respective species. Although the tables with these numbers are symmetric along the diagonal, the complete tables are presented here for easy reading.

**Input Parameters**

Supplementary Table 1 shows the settings used to run the SNIT pipeline for each of the five species.

Supplementary Table 1. Input parameters used to run the SNIT pipeline

| **Parameter** | **Value** |
| --- | --- |
| (MUMmer) min. cluster length | 100 |
| (MUMmer) min. exact match | 50 |
| (MUMmer) max.gap | 49 |
| min. large indel size | 50 |
| min. conserved flank length | 50 |
| min. distance from edges | 100 |
| mask tandem repeats | selected |
| TRF flags | 2 5 5 80 10 50 500 –h |

***Bacillus anthracis***

To evaluate the accuracy for *B. anthracis*, we used the phylogeny presented in Van Ert, *et al.,* 2007.

Supplementary Table 2. Details of the 7 *B. anthracis* strains used in the analysis

| **Strain** | **NCBI Accession Number** |
| --- | --- |
| Ames | NC_003997.3 |
| A0155 | NZ_AAEO01000000 |
| CNEVA | NZ_AAEN00000000 |
| Kruger B | NZ_AAEQ00000000 |
| WNA (USA 6153) | NZ_AAER00000000 |
| Vollum | NZ_AAEP00000000 |
| Australia 94 | NZ_AAES00000000 |

Supplementary Table 3. Number of SNP/indel loci in each pair of *B. anthracis* genomes that have different alleles

| **Strain** | **anthr_ames** | **anth_a0155** | **cneva_9066** | **ba_usa6153** | **ba_austr94** | **ba_krugerb** | **ba_vollum** |
| --- | --- | --- | --- | --- | --- | --- | --- |
| **anthr_ames** | 0 | 2227 | 1433 | 577 | 468 | 1842 | 666 |
| **anth_a0155** | 2227 | 0 | 2114 | 2154 | 2254 | 2580 | 2203 |
| **cneva_9066** | 1433 | 2114 | 0 | 1349 | 1461 | 852 | 1412 |
| **ba_usa6153** | 577 | 2154 | 1349 | 0 | 577 | 1780 | 546 |
| **ba_austr94** | 468 | 2254 | 1461 | 577 | 0 | 1881 | 672 |
| **ba_krugerb** | 1842 | 2580 | 852 | 1780 | 1881 | 0 | 1830 |
| **ba_vollum** | 666 | 2203 | 1412 | 546 | 672 | 1830 | 0 |

Note: The Western North America (WNA) strain is named usa6153 here.

**Accuracy computations**

Supplementary Table 4. Comparison of closest neighbors with published results

| **Strain** | **Closest Neighbor from Van Ert, *et al.,* 2007** | **Closest Neighbor from SNIT (no of SNPs)** | **Next Closest Neighbor (no of SNPs)** |
| --- | --- | --- | --- |
| **A0155** | CNEVA or Vollum | CNEVA (2114) | WNA (2154) |
| **CNEVA** | KrugerB | KrugerB (852) | WNA (1349) |
| **KrugerB** | CNEVA | CNEVA (852) | WNA (1780) |
| **Vollum** | Aust94 or WNA | WNA (546) | Ames (666) |
| **Aust94** | Ames | Ames (468) | WNA (577) |
| **Ames** | Aust94 | Aust94 (468) | WNA (577) |
| **WNA (USA 6153)** | Vollum | Vollum (546) | Ames (577) |

**Accuracy: 7/7 => 100%**

***Francisella tularensis***

To evaluate the accuracy for *F. tularensis*, we used the phylogeny presented in Champion, *et al.,* 2009 and Larsson, *et al.,* 2009.

Supplementary Table 5. Details of the 11 *F. tularensis* strains used in the analysis

| **Strain** | **NCBI Accession Number** |
| --- | --- |
| mediasiatica FSC147 | NC_010677.1 |
| tularensis Schu 4 | NC_006570.1 |
| tularensis WY96-3418 | NC_009257.1 |
| tularensis FSC198 | NC_008245.1 |
| holarctica FTA | NC_009749.1 |
| holarctica (LVS) | NC_007880.1 |
| holarctica OSU18 | NC_008369.1 |
| tularensis FSC033 | AAYE010000[01-15].1 |
| holarctica 257 | AAUD010000[01-31].1 |
| holarctica FSC022 | AAYD010000[01-19].1 |
| holarctica FSC200 | NZ_AASP010000[01-39].1 |

Supplementary Table 6. Number of SNP/indel loci in each pair of *F. tularensis* genomes that have different alleles

| **Strain** | **FSC147** | **SCHU4** | **WY96** | **FSC198** | **FTA** | **LVS** | **OSU18** | **FSC033** | **FSC257** | **FSC022** | **FSC200** |
| --- | --- | --- | --- | --- | --- | --- | --- | --- | --- | --- | --- |
| **FSC147** | 0 | 3673 | 3193 | 3670 | 4419 | 4391 | 4425 | 3657 | 4432 | 4120 | 4669 |
| **SCHU4** | 3673 | 0 | 2639 | 19 | 4952 | 4897 | 4930 | 133 | 4982 | 4577 | 5149 |
| **WY96** | 3193 | 2639 | 0 | 2632 | 4324 | 4269 | 4307 | 2631 | 4357 | 3963 | 4560 |
| **FSC198** | 3670 | 19 | 2632 | 0 | 4955 | 4900 | 4933 | 128 | 4985 | 4580 | 5148 |
| **FTA** | 4419 | 4952 | 4324 | 4955 | 0 | 524 | 566 | 4953 | 644 | 2756 | 1061 |
| **LVS** | 4391 | 4897 | 4269 | 4900 | 524 | 0 | 531 | 4900 | 176 | 2715 | 638 |
| **OSU18** | 4425 | 4930 | 4307 | 4933 | 566 | 531 | 0 | 4930 | 655 | 2730 | 1062 |
| **FSC033** | 3657 | 133 | 2631 | 128 | 4953 | 4900 | 4930 | 0 | 4987 | 4571 | 5147 |
| **FSC257** | 4432 | 4982 | 4357 | 4985 | 644 | 176 | 655 | 4987 | 0 | 2817 | 754 |
| **FSC022** | 4120 | 4577 | 3963 | 4580 | 2756 | 2715 | 2730 | 4571 | 2817 | 0 | 3115 |
| **FSC200** | 4669 | 5149 | 4560 | 5148 | 1061 | 638 | 1062 | 5147 | 754 | 3115 | 0 |

**Accuracy computations**

Supplementary Table 7. Comparison of closest neighbors with published results

| **Strain** | **Closest Neighbor from Champion, et al., 2009** | **Closest Neighbor from SNIT (no of SNPs)** | **Next Closest Neighbor (no of SNPs)** |
| --- | --- | --- | --- |
| **FTA** | LVS or FSC200 or FSC257 | LVS (524) | OSU18 (566) |
| **LVS** | FSC257 or FSC200 | FSC257 (176) | FTA (524) |
| **OSU18** | FTA or LVS | LVS (531) | FTA (566) |
| **FSC022** | LVS, FTA or OSU18 | LVS (2715) | OSU18 (2730) |
| **FSC147** | WY96 | WY96 (3193) | FSC033 (3657) |
| **SCHU4** | FSC198 | FSC198 (19) | FSC033 (133) |
| **FSC033** | SCHU4 or FSC198 | FSC198 (128) | SCHU4 (133) |
| **WY96** | FSC033 or SCHU4 or FSC198 | FSC033 (2631) | FSC198 (2632) |
| **FSC257** | LVS | LVS (176) | FTA (644) |
| **FSC200** | LVS | LVS (638) | FSC257 (754) |
| **FSC198** | SCHU4 | SCHU4 (19) | FSC033 (128) |

**Accuracy: 11/11 => 100%**

***Shigella flexneri***

To evaluate the accuracy for *S. flexneri*, we used the phylogeny presented in Ye, *et al*., 2010.

Supplementary Table 8. Details of the 4 *S. flexneri* strains used in the analysis

| **Strain** | **NCBI Accession Number** |
| --- | --- |
| 2a str. 301 | NC_004337.1 |
| 2a str. 2457T | NC_004741.1 |
| 5 str. 8401 | NC_008258.1 |
| 2002017 (strain X) | CP001383.1 |

Supplementary Table 9. Number of SNP/indel loci in each pair of *S. flexneri* genomes that have different alleles

| **Strain** | **301** | **2457T** | **8401** | **20022017** |
| --- | --- | --- | --- | --- |
| **301** | 0 | 682 | 3432 | 863 |
| **2457T** | 682 | 0 | 3506 | 627 |
| **8401** | 3432 | 3506 | 0 | 3663 |
| **20022017** | 863 | 627 | 3663 | 0 |

**Accuracy computations**

Supplementary Table 10. Comparison of closest neighbors with published results

| **Strain** | **Closest Neighbor from Ye, *et al.*, 2010** | **Closest Neighbor from SNIT (no of SNPs)** | **Next Closest Neighbor (no of SNPs)** |
| --- | --- | --- | --- |
| **301** | 2457t or X | 2457t (682) | X (863) |
| **2457t** | X | X (627) | 301 (682) |
| **8401** | All three are equally likely | 301 (3432) | 2457t (3506) |
| **20022017 (X)** | 2457t | 2457t (627) | 301 (863) |

**Accuracy: 4/4 => 100%**

***Burkholderia mallei***

To evaluate the accuracy for *B. mallei*, we used the phylogeny presented in Pearson, *et al.,* 2009.

Supplementary Table 11. Details of the 10 *B. mallei* strains used in the analysis

| **Strain** | **NCBI Accession Number** |
| --- | --- |
| ATCC 23344 | NC_006348.1, NC_006349.2 |
| NCTC 10229 | NC_008835.1, NC_008836.1 |
| NCTC 10247 | NC_009079.1, NC_009080.1 |
| SAVP1 | NC_008784.1, NC_008785.1 |
| 2002721280 | NZ_AANX00000000 |
| ATCC 10399 | NZ_AAHN00000000 |
| FMH | NZ_AAIQ00000000 |
| GB8 Horse 4 | NZ_AAHO00000000 |
| JHU | NZ_AAIR00000000 |
| PRL-20 | NZ_AAZP00000000 |

Supplementary Table 12. Number of SNP/indel loci in each pair of *B. mallei* genomes that have different alleles

| **Strain** | **atcc**  **23344** | **nctc**  **10229** | **nctc**  **10247** | **savp1** | **2002721280** | **atcc10399** | **fmh** | **gb8** | **jhu** | **prl20** |
| --- | --- | --- | --- | --- | --- | --- | --- | --- | --- | --- |
| **atcc23344** | 0 | 520 | 513 | 382 | 529 | 336 | 12 | 14 | 17 | 427 |
| **nctc10229** | 520 | 0 | 109 | 477 | 180 | 567 | 518 | 518 | 525 | 534 |
| **nctc10247** | 513 | 109 | 0 | 470 | 168 | 556 | 511 | 511 | 518 | 527 |
| **savp1** | 382 | 477 | 470 | 0 | 485 | 428 | 380 | 380 | 387 | 117 |
| **2002721280** | 529 | 180 | 168 | 485 | 0 | 579 | 527 | 527 | 534 | 542 |
| **atcc10399** | 336 | 567 | 555 | 428 | 519 | 0 | 334 | 336 | 341 | 471 |
| **fmh** | 12 | 518 | 511 | 380 | 527 | 334 | 0 | 12 | 17 | 426 |
| **gb8** | 14 | 518 | 511 | 380 | 527 | 336 | 12 | 0 | 19 | 428 |
| **jhu** | 17 | 525 | 518 | 387 | 534 | 341 | 17 | 19 | 0 | 433 |
| **prl20** | 427 | 534 | 527 | 117 | 542 | 471 | 426 | 428 | 433 | 0 |

Supplementary Table 13. Comparison of closest neighbors with published results

| **Strain** | **Closest Neighbor from Pearson, *et al*., 2009** | **Closest Neighbor from SNIT (no of SNPs)** | **Next Closest Neighbor (no of SNPs)** |
| --- | --- | --- | --- |
| **ATCC23344** | FMH, GB8 or JHU | FMH (12) | JHU (14) |
| **NCTC10229** | NCTC10247 | NCTC10247 (109) | 2002721280 (180) |
| **NCTC10247** | NCTC10229 | NCTC10229 (109) | 2002721280 (168) |
| **SAVP1** | PRL20 | PRL20 (117) | FMH (380), GB8 (380) |
| **2002721280** | NCTC 10247 or 10229 | NCTC10247 (168) | NCTC10229 (180) |
| **ATCC10399** | ATCC 23344, JHU, GB8, FMH | FMH (334) | ATCC23344 (336), GB8 (336) |
| **FMH** | JHU, GB8, ATCC23344 | ATCC23344 (12), GB8 (12) | JHU (17) |
| **GB8** | JHU, FMH, ATCC23344 | FMH (12) | ATCC23344 (14) |
| **JHU** | FMH, GB8, ATCC23344 | ATCC23344 (17), FMH (17) | GB8 (19) |
| **Prl20** | SAVP1 | SAVP1 (117) | FMH (426) |

**Accuracy: 10/10 => 100%**

***Burkholderia pseudomallei***

To evaluate the accuracy for *B. pseudomallei*, we used the phylogeny presented in Pearson, *et al.,* 2009.

Supplementary Table 14. Details of the 20 *B. pseudomallei* strains used in the analysis

| **Strain** | **NCBI Accession Number** |
| --- | --- |
| 668 | NC_009074.1, NC_009075.1 |
| DM98 | NZ_ABBI0100[0001-2371] |
| 1655 | NZ_AAHR00000000 |
| 305 | NZ_AAYX010000[01-36].1 |
| NCTC13177 | NZ_ABBQ0100[0001-1077].1 |
| K96243 | NC_006350.1, NC_006351.1 |
| 1106a | NC_009076.1, NC_009078.1 |
| 1106b | NZ_AAMB00000000 |
| 112 | NZ_ABBP0100[0001-1274].1 |
| 14 | NZ_ABBJ0100[0001-1888].1 |
| 1710a | NZ_AAHS00000000 |
| 1710b | NC_007434.1, NC_007435.1 |
| 406e | VZ_AAMM00000000 |
| 7894 | NZ_ABBO0100[0001-1568].1 |
| 91 | NZ_ABBK0100[0001-1690].1 |
| 9 | NZ_ABBL010000[01-70].1 |
| B7210 | NZ_ABBN0100[0001-1424].1 |
| BCC215 | NZ_ABBR0100[0001-1030].1 |
| S13 | NZ_AAHW00000000 |
| Pasteur 52237 | NZ_AAHV00000000 |

Supplementary Table 15. Number of SNP/indel loci in each pair of *B. pseudomallei* genomes that have different alleles

| **Strain** | **668** | **dm98** | **Bp1655** | **305** | **nctc13177** | **k96243** | **1106a** | **1106b** | **112** | **Bp14** | **1710a** | **1710b** | **406e** | **7894** | **Bp91** | **Bp9** | **b7210** | **bcc215** | **pasteur** | **s13** |
| --- | --- | --- | --- | --- | --- | --- | --- | --- | --- | --- | --- | --- | --- | --- | --- | --- | --- | --- | --- | --- |
| **668** | 0 | 9222 | 9443 | 9666 | 9555 | 9374 | 9359 | 9358 | 9410 | 9184 | 9273 | 9265 | 9366 | 9384 | 9343 | 9337 | 9477 | 9522 | 9364 | 9397 |
| **dm98** | 9222 | 0 | 9316 | 9982 | 9761 | 9536 | 9591 | 9597 | 9548 | 9530 | 9475 | 9463 | 9515 | 9572 | 9498 | 9519 | 9734 | 9720 | 9494 | 9605 |
| **Bp1655** | 9443 | 9316 | 0 | 9849 | 9711 | 9362 | 9364 | 9370 | 9459 | 9351 | 9312 | 9304 | 9361 | 9390 | 9416 | 9371 | 9611 | 9537 | 9214 | 9503 |
| **305** | 9666 | 9982 | 9849 | 0 | 9641 | 9945 | 9927 | 9932 | 9976 | 9818 | 9785 | 9775 | 9915 | 9924 | 9953 | 9862 | 10162 | 10089 | 9894 | 9915 |
| **nctc13177** | 9555 | 9761 | 9711 | 9641 | 0 | 9708 | 9703 | 9705 | 9848 | 9683 | 9634 | 9622 | 9844 | 9876 | 9756 | 9761 | 9860 | 10023 | 9593 | 9730 |
| **k96243** | 9374 | 9536 | 9362 | 9945 | 9708 | 0 | 7502 | 7509 | 7553 | 7720 | 6991 | 6983 | 7660 | 8166 | 2036 | 7781 | 7845 | 8483 | 7087 | 7994 |
| **1106a** | 9359 | 9591 | 9364 | 9927 | 9703 | 7502 | 0 | 23 | 7504 | 7410 | 7052 | 7042 | 7260 | 8260 | 7515 | 7208 | 3195 | 8538 | 6927 | 5950 |
| **1106b** | 9358 | 9597 | 9370 | 9932 | 9705 | 7509 | 23 | 0 | 7514 | 7416 | 7055 | 7043 | 7265 | 8265 | 7522 | 7214 | 3214 | 8543 | 6935 | 5961 |
| **112** | 9410 | 9548 | 9459 | 9976 | 9848 | 7553 | 7504 | 7514 | 0 | 7369 | 6573 | 6557 | 6912 | 8350 | 7611 | 7292 | 7791 | 8648 | 6572 | 7806 |
| **Bp14** | 9184 | 9530 | 9351 | 9818 | 9683 | 7720 | 7410 | 7416 | 7369 | 0 | 6734 | 6724 | 7210 | 8274 | 7733 | 7085 | 7670 | 8588 | 6879 | 7682 |
| **1710a** | 9273 | 9475 | 9312 | 9785 | 9634 | 6991 | 7052 | 7055 | 6573 | 6734 | 0 | 32 | 6458 | 8115 | 6995 | 6771 | 7349 | 8450 | 4104 | 7579 |
| **1710b** | 9265 | 9463 | 9304 | 9775 | 9622 | 6983 | 7042 | 7043 | 6557 | 6724 | 32 | 0 | 6448 | 8105 | 6987 | 6765 | 7339 | 8438 | 4092 | 7567 |
| **406e** | 9366 | 9515 | 9361 | 9915 | 9844 | 7660 | 7260 | 7265 | 6912 | 7210 | 6458 | 6448 | 0 | 8236 | 7653 | 6987 | 7637 | 8522 | 6562 | 7373 |
| **7894** | 9384 | 9572 | 9390 | 9924 | 9876 | 8166 | 8260 | 8265 | 8350 | 8274 | 8115 | 8105 | 8236 | 0 | 8175 | 8237 | 8336 | 3229 | 8157 | 8287 |
| **Bp91** | 9343 | 9498 | 9416 | 9953 | 9756 | 2036 | 7515 | 7522 | 7611 | 7733 | 6995 | 6987 | 7653 | 8175 | 0 | 7679 | 7808 | 8544 | 7070 | 8010 |
| **Bp9** | 9337 | 9519 | 9371 | 9862 | 9761 | 7781 | 7208 | 7214 | 7292 | 7085 | 6771 | 6765 | 6987 | 8237 | 7679 | 0 | 7732 | 8648 | 6712 | 7739 |
| **b7210** | 9477 | 9734 | 9611 | 10162 | 9860 | 7845 | 3195 | 3214 | 7791 | 7670 | 7349 | 7339 | 7637 | 8336 | 7808 | 7732 | 0 | 8647 | 7230 | 6189 |
| **bcc215** | 9522 | 9720 | 9537 | 10089 | 10023 | 8483 | 8538 | 8543 | 8648 | 8588 | 8450 | 8438 | 8522 | 3229 | 8544 | 8648 | 8647 | 0 | 8467 | 8638 |
| **pasteur** | 9364 | 9494 | 9214 | 9894 | 9593 | 7087 | 6927 | 6935 | 6572 | 6879 | 4104 | 4092 | 6562 | 8157 | 7070 | 6712 | 7230 | 8467 | 0 | 7574 |
| **S13** | 9397 | 9605 | 9503 | 9915 | 9730 | 7994 | 5950 | 5961 | 7806 | 7682 | 7579 | 7567 | 7373 | 8287 | 8010 | 7739 | 6189 | 8638 | 7574 | 0 |

**Accuracy computation**

Supplementary Table 16. Comparison of closest neighbors with published results. Entries that differ from published results are highlighted in red.

| **Strain** | **Closest Neighbor from Pearson *et al*., 2009** | **Closest Neighbor from SNIT (no of SNPs)** | **Next Closest Neighbor (no of SNPs)** |
| --- | --- | --- | --- |
| **668** | Not clear | Bp14 (9184) | dm98 (9222) |
| **dm98** | Bp1655 | 668 (9222) | Bp1655 (9316) |
| **Bp1655** | DM98 | pasteur (9214) | 1710b (9304) |
| **Bp305** | NCTC13177 | NCTC13177 (9641) | 1710b (9775) |
| **NCTC13177** | Bp305 | 668 (9555) | pasteur (9593) |
| **K96243** | Bp91 | Bp91(2036) | pasteur (7087) |
| **1106a** | 1106b | 1106b (23) | S13 (5950) |
| **1106b** | 1106a | 1106a (23) | S13 (5961) |
| **112** | Pasteur, 1710a, or 1710b | 1710b (6557) | pasteur (6572) |
| **Bp14** | Bp9 | 1710b (6724) | 1710a (6734) |
| **1710a** | 1710b | 1710b (32) | Pasteur (4104) |
| **201710b** | 1710a | 1710a (32) | Pasteur (4092) |
| **Bp406e** | Bp112, Pasteur, or 1710 | 1710b (6448) | 1710a (6458) |
| **Bp7894** | BCC215 | BCC215 (3229) | 1710b (8105) |
| **Bp91** | K96243 | K96243 (2036) | 1710b (6987) |
| **Bp9** | Bp14 | pasteur (6712) | 1710b (6765) |
| **B7210** | 1106 a or b | 1106a (3195) | 1106b (3214) |
| **Bcc215** | Bp7894 | Bp7894 (3229) | 1710b (8438) |
| **Pasteur** | 1710 a or b | 1710b (4092) | 1710a (4104) |
| **S13** | 1106a or 1106b or B7210 | 1106a (5950) | 1106b (5961) |

**Accuracy: 15/20 => 75%; error: 25%**

**References**

Champion, M.D., Zeng, Q., Nix, E.B., Nano, F.E., Keim, P., Kodira, C.D., Borowsky, M., Young, S., Koehrsen, M., Engels, R., Pearson, M., Howarth, C., Larson, L., White, J., Alvarado, L., Forsman, M., Bearden, S.W., Sjostedt, A., Titball, R., Michell, S.L., Birren, B. and Galagan, J. (2009) Comparative genomic characterization of Francisella tularensis strains belonging to low and high virulence subspecies, *PLoS Pathog*, **5**, e1000459.

Larsson, P., Elfsmark, D., Svensson, K., Wikstrom, P., Forsman, M., Brettin, T., Keim, P. and Johansson, A. (2009) Molecular evolutionary consequences of niche restriction in Francisella tularensis, a facultative intracellular pathogen, *PLoS Pathog*, **5**, e1000472.

Pearson, T., Giffard, P., Beckstrom-Sternberg, S., Auerbach, R., Hornstra, H., Tuanyok, A., Price, E.P., Glass, M.B., Leadem, B., Beckstrom-Sternberg, J.S., Allan, G.J., Foster, J.T., Wagner, D.M., Okinaka, R.T., Sim, S.H., Pearson, O., Wu, Z., Chang, J., Kaul, R., Hoffmaster, A.R., Brettin, T.S., Robison, R.A., Mayo, M., Gee, J.E., Tan, P., Currie, B.J. and Keim, P. (2009) Phylogeographic reconstruction of a bacterial species with high levels of lateral gene transfer, *BMC Biol*, **7**, 78.

Van Ert, M.N., Easterday, W.R., Huynh, L.Y., Okinaka, R.T., Hugh-Jones, M.E., Ravel, J., Zanecki, S.R., Pearson, T., Simonson, T.S., U'Ren, J.M., Kachur, S.M., Leadem-Dougherty, R.R., Rhoton, S.D., Zinser, G., Farlow, J., Coker, P.R., Smith, K.L., Wang, B., Kenefic, L.J., Fraser-Liggett, C.M., Wagner, D.M. and Keim, P. (2007) Global genetic population structure of Bacillus anthracis, *PLoS ONE*, **2**, e461.

Ye, C., Lan, R., Xia, S., Zhang, J., Sun, Q., Zhang, S., Jing, H., Wang, L., Li, Z., Zhou, Z., Zhao, A., Cui, Z., Cao, J., Jin, D., Huang, L., Wang, Y., Luo, X., Bai, X., Wang, P., Xu, Q. and Xu, J. (2010) Emergence of a new multidrug-resistant serotype X variant in an epidemic clone of Shigella flexneri, *J Clin Microbiol*, **48**, 419-426.
